# The Use of a PVDF Array to Measure the Stress Field Inside an Elastic Material

**DOI:** 10.3390/s23042144

**Published:** 2023-02-14

**Authors:** Ming Jin, David Matthews, Ning Wang, Jie Pan

**Affiliations:** Department of Mechanical Engineering, University of Western Australia, Crawley, WA 6009, Australia

**Keywords:** embedded PVDF, elastic material, sensor array, FEM

## Abstract

This paper reports a series of experimental and modeling investigations on two piezoelectric arrays made of polyvinylidene fluoride (PVDF) films. They were embedded inside rubber cylinders and used to directly measure the internal stresses generated by various external excitations applied to the top surface of the cylinder. Corresponding finite element (FE) models were established to reveal the relationship between the PVDF output and the stress field applied to it. This research improves the understanding of the output mechanism of the embedded PVDF and provides useful information for the design of PVDF sensors.

## 1. Introduction

As a novel piezoelectric material, polyvinylidene fluoride (PVDF) has broad application prospects. Compared with traditional ceramic sensors, PVDF has many unique advantages, such as high sensitivity, a light weight, and corrosion resistance. As a result, PVDF sensors have been widely used for many vibro-acoustic measurements [1,2,3]. For many of these, the PVDF sensor is mounted directly on the surface of the structure, however, due to its nonobtrusive nature, it is also possible to embed the films inside materials to measure the internal stress field with minimal effect on the stress field itself. This makes PVDF suitable for use as an intrinsic sensor in difficult environments such as inside concrete structures [4], inside thrust bearings [5], and underwater [6,7]. However, the output of embedded PVDF is affected by many factors, so a thorough understanding of its working mechanism is a challenging task.

**Figure 1:**
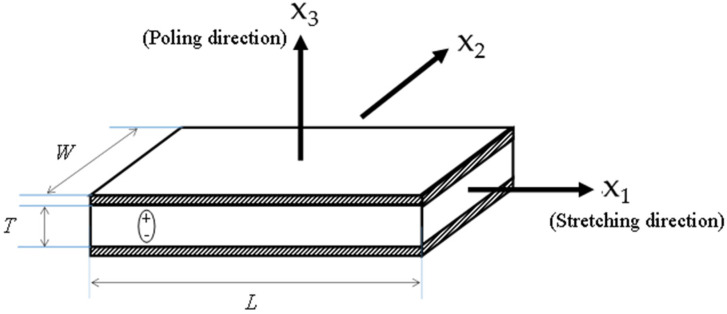
Schematic of the PVDF film used. In the manufacturing process, the film has been polarized in the x_3_ direction and stretched in the x_1_ direction.

Compared with single-axis sensors such as accelerometers, the output of a PVDF film is simultaneously affected by the stresses in the three directions applied to it, and the open-circuit voltage output is expressed as [8]:

(1) $$V_{PVDF}=T(g_{33}{\bar{\tau }}_{33}^{}+g_{32}{\bar{\tau }}_{22}^{}+g_{31}{\bar{\tau }}_{11}^{})$$

where *T* is the thickness of the PVDF film, *g*_3*i*_ is the piezoelectric voltage constant of the PVDF in its *x_i_* direction, and $\bar{\tau_{ii}}$ is the averaged volume stress in the PVDF in the *x_i_* direction. The three directions of the PVDF sensor are determined by its manufacturing process (See Figure 1). In addition, most commercially available PVDF films have anisotropic piezoelectric voltage constants which further increase the complexity of the PVDF output. This is compounded by the elastic properties of the PVDF itself whose output is not only determined by its own mechanical and piezoelectric properties but also by the mechanical properties of the material around it. As a result, understanding the output of PVDF attached to various structures can be difficult and often shows some “counter-intuitive” results. For example, Du [4] reported an experimental work using embedded PVDF sensors to monitor the internal stress of a concrete cylinder excited by an impact force. The results showed that the PVDF sensor farthest from the excitation had the larger output. Shapiro [9] used embedded PVDF to track the deformation of a planar hyper-flexible beam, and the experiment demonstrated that the sensitivity of PVDF sensors located at certain locations of the beam was much lower than others. Without the thorough understanding of the working mechanisms of embedded PVDF, its output lacks a clear physical meaning. Therefore, in many current applications, embedded PVDF can only be used as an auxiliary to other sensors [10,11], and its output can only be analyzed qualitatively [12]. This difficulty in interpreting the output of embedded PVDF sensors has limited its use as a sensor. To address this issue, in this paper, a series of experimental and modeling studies were performed to improve the understanding of the physical mechanisms responsible for the observed voltage output of a PVDF film embedded in an elastic material. Two rubber cylinders with different arrangements of embedded PVDF arrays were tested, and corresponding finite element (FE) models were established. Firstly, the accuracy of the FE models was verified by comparing the experimental and simulated results. Then, through the analysis of the FE models, the mechanism of the embedded PVDF was further understood, giving good agreement with the experimental results.

## 2. Vertical PVDF Array

### 2.1. Experimental Description of Cylinder 1

**Figure 2:**
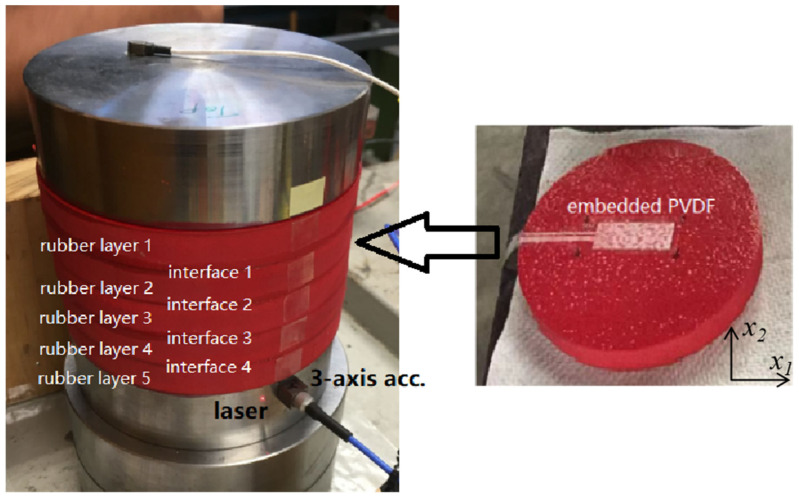
The 5-layer rubber cylinder with embedded longitudinal PVDF array.

Figure 2 shows the first rubber cylinder constructed using five thin rubber layers with two steel blocks at the two ends. The diameter of the cylinder is 93 mm, and the thickness of each rubber layer is 10 mm. The thickness of each steel block is 25 mm. A longitudinal sensor array consists of four MEAS FLDT-28K PVDF films that were embedded into the rubber interfaces from interface 1 to interface 4 as shown in Figure 2 and located at the mid-line of the cylinder. The electrode area of the sensor is 30 mm × 12 mm, and its thickness is 28 μm. All items were glued by Solufix 14 rubber glue whose mechanical properties are similar to the rubber after dried.

During the test, the cylinder was excited by an impact hammer at the center of the top steel block in the normal direction. In addition to the PVDF array, a 3-axis accelerometer and a laser vibrometer were also used to measure the vibration of the cylinder. This allowed a direct comparison of the PVDF output and surface vibration of the structure. The outputs of all sensors were digitized using a B&K LAN-XI data acquisition system and analyzed by PULSE data analysis software. 

### 2.2. Results of Cylinder 1 and Discussion

**Figure 3:**
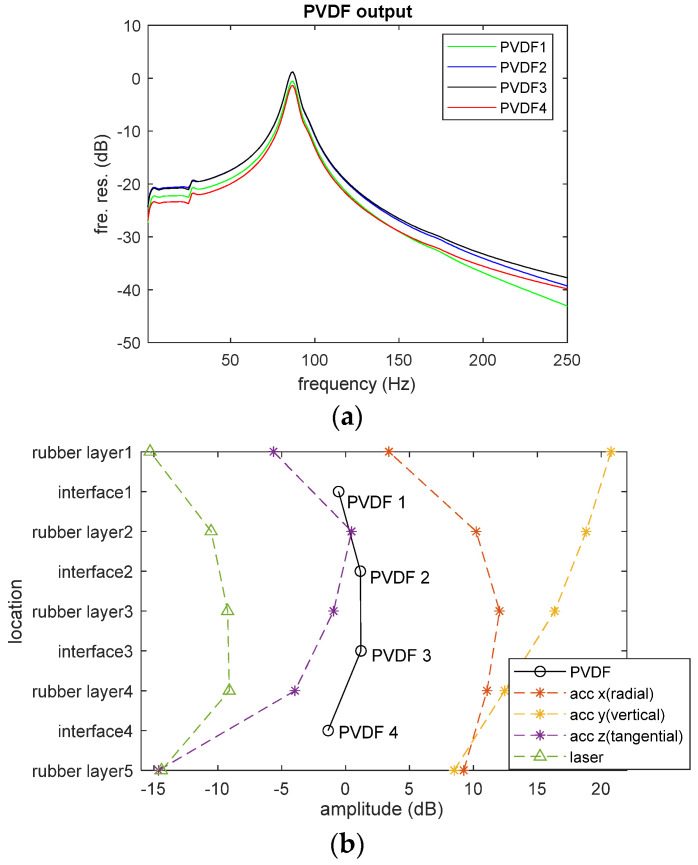
(**a**) The frequency responses of the embedded PVDF array and (**b**) output distribution of the sensors at 87 Hz.

When the rubber cylinder was excited by the hammer at the center of the top mass block, all the sensors only showed a peak at 87 Hz within the measured frequency range (up to 1600 Hz), which should be corresponding to the natural frequency of the axial mode of the cylinder [13]. Figure 3a displays the response of the PVDF array. The y axis represents the frequency response of the PVDF output voltage with respect to the impact force. Although it might be expected that the magnitude of this peak should decay from the top to the bottom of the cylinder, it was found that PVDF 2 at interface 2 and PVDF 3 at interface 3, which were attached to the middle of the cylinder, had the largest outputs. At 87 Hz, this difference is approximately 1.8 dB (23%). Figure 3b also shows the output distribution of the accelerometer and laser vibrometer at the same frequency. The y axis is the measurement location from the top to the bottom of the cylinder. Acc x, acc y, and acc z represent the radial, vertical, and tangential accelerations measured on the surface of the cylinder by the 3-axis accelerometer, respectively. The laser vibrometer collected the radial velocity on the surface of the cylinder. It is clear that only the vertical acceleration, measured by the y axis of the 3-axis accelerometer, monotonically decreases down the cylinder. All other sensor outputs have a maximum at the middle of the cylinder and minimum at the two ends. Since both the laser vibrometer and x and z axes of the 3-axis accelerometer measured the in-plane vibration for the cylinder, it is necessary to include these contributions when considering the output of embedded PVDF films. 

**Figure 4:**
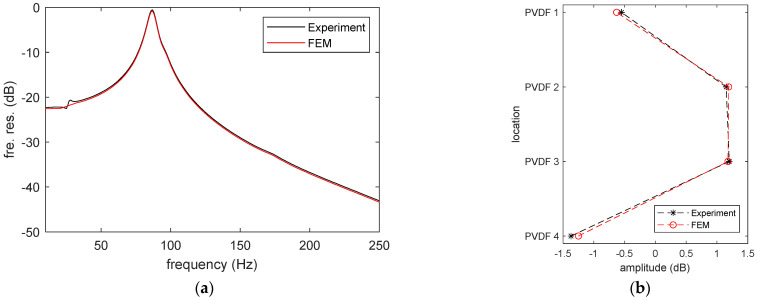
Comparison between the FE model and experiment: (**a**) frequency response of PVDF 1 and (**b**) output distribution of the PVDF array at 87 Hz.

To explain the experimental observations, the interaction between the embedded PVDF films and the elastic material was simulated using the COMSOL multiphysics model. The governing principles include the force equilibrium equations in solid mechanics and the constitutive equations of the linear elastic and piezoelectric material. Considering the excitation used in the experiment is an impact force and its amplitude is relatively small (about 100 N), the deformation of the rubber should be mainly due to its elastic property [14]. Therefore, the rubber is modeled as a pure elastic material and its viscoelastic property is not considered. The elastic material and PVDF films are assumed to be perfectly bonded to each other, therefore the continuity of displacement and strain and the equilibrium of stress are applied at the interface between them. The top surface of the film is set as the voltage terminal, while the bottom is connected to the ground. The voltage value is extracted directly without any other circuit component, in order to simulate the open-circuit condition. The current or the free charge on the top surface is equal to zero at all times. The mesh size is determined through the convergence study. The mechanical properties of the PVDF and rubber used in the FE model were obtained experimentally, and the piezoelectric properties of PVDF were obtained from the product manual [15]. These parameters are listed in Table 1. In the FE model, the bottom steel block was ignored because it does not affect the simulation in the low frequency range. Figure 4 compares the simulated results with the experimental data. As can be seen, there is a good agreement for the output of PVDF 1 with the RMS difference of each PVDF element less than 0.2 dB within the interested frequency range (1 Hz < f < 250 Hz). The difference is mainly due to some experimental error. For example, the small peak at 28 Hz measured by the PVDF in Figure 4a is the shear mode of the rubber cylinder due to the slight off-central excitation in the experiment. Meanwhile, Figure 4b indicates that the output distribution of the PVDF array calculated by the FE model also displays the same trend as that observed in the experiment. 

**Figure 5:**
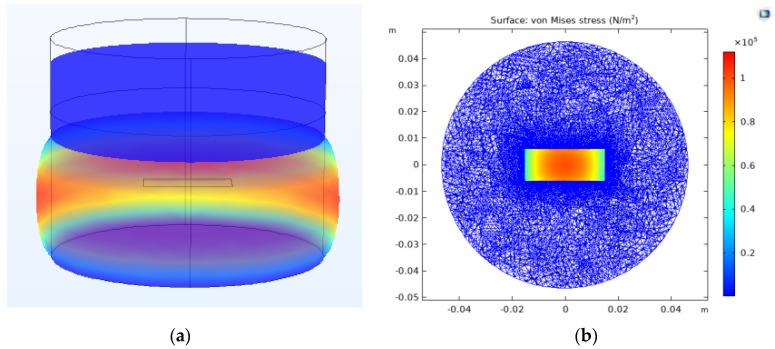
FE simulation (**a**) the in-plane strain distribution of the cylinder at 87 Hz and (**b**) the internal in-plane stress field of the cylinder.

After the FE model was verified, it was used to investigate the output mechanism of the PVDF array inside the rubber structure. Figure 5a shows the in-plane strain distribution of the rubber cylinder at 87 Hz, which is its axial mode. When the normal (vertical) excitation is applied at the top of the rubber cylinder, it not only generates the vertical deformation of the rubber but also leads to in-plane deformation (strain) due to the Poisson effect. Due to the clamped boundaries at the steel interfaces, the in-plane deformation at the two ends of rubber cylinder is zero and gradually increases away from the ends reaching a maximum in the middle. The large in-plane strain of the rubber is due to its large internal in-plane stress. Because the output of PVDF is controlled by all three principal normal stresses applied to it, the PVDF element close to the middle part of the cylinder will have a larger output if its output is dominated by the in-plane stress. 

Table 2 shows the averaged volume stresses applied on each PVDF element estimated from the FE model. It is clear that the in-plane stresses ($\bar{\tau_{11}}$ and $\bar{\tau_{22}}$) are much larger than the vertical stress ($\bar{\tau_{33}}$). As a result, the output of the PVDF is mainly controlled by the in-plane stress. For example, substituting the stresses of PVDF 2 into Equation (1), the contribution of $\bar{\tau_{11}}$, $\bar{\tau_{22}}$, and $\bar{\tau_{33}}$ on its overall output are 92.0%, 7.1%, and 0.9%, respectively. It is, therefore, clear that the output of the PVDF film is dominated by the stress in the x_1_ direction. Secondly, the stresses applied to PVDF 2 and PVDF 3 are always larger than those applied to PVDF 1 and PVDF 4. This explains the output distribution observed in the experiment shown in Figure 3b. This finding indicates that the output of embedded PVDF is significantly affected by the properties of the elastic material around it. Further research indicated that softer material with higher Poisson ratio can enhance the output of the embedded PVDF [16]. Another interesting phenomenon observed from the FE simulation is that the internal stress field has a sharp jump at the interface between rubber and PVDF [see Figure 5b]. This stress concentration is due to the huge strength difference between them. In long-term use, stress concentration may cause some potential problems, such as accelerated glue peeling and PVDF fracture [17]. Although these issues do not affect measurements in the laboratory environment, they should be considered in practical long-term applications of embedded PVDF. 

## 3. Horizontal PVDF Array

### 3.1. Experimental Description of Cylinder 2

**Figure 6:**
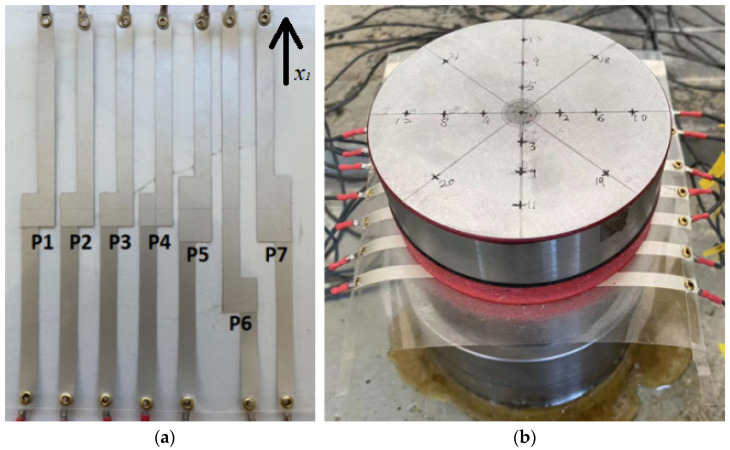
(**a**) The embedded PVDF array and (**b**) the 2-layer rubber cylinder.

**Figure 7:**
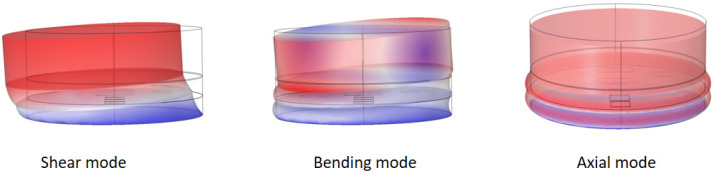
The three modes of the rubber cylinder computed by FE model.

In the first 5-layer rubber cylinder discussed above, it was demonstrated that the in-plane stress of the elastic material significantly affects the output of the embedded PVDF. To further investigate this issue, another two-layer rubber cylinder with a horizontal (in-plane) PVDF array was built. With cylinder 2, the size of the rubber layers and steel blocks used is the same as the previous rig. The PVDF array consists of 7 PVDF elements. The previous simulation demonstrated that small PVDF film leads to stress concentration around it [see Figure 5b]. To avoid this issue, rather than using 7 individual PVDF films, the PVDF array was made using one sheet of PVDF film purchased from Measurement Specialties. The thickness of the PVDF sheet was 0.11 mm and was supplied with silver electrodes screen-printed on both sides. Individual electrodes were etched on both sides of the film using a stencil and isopropyl alcohol. The PVDF array is shown in Figure 6a. The x_1_ direction of PVDF is perpendicular to the array. The transparent material is the PVDF sheet without electrode. The silver squares/rectangles are the active PVDF elements which have electrodes on both surfaces. The silver strips connected to the elements are the “wires” extending in opposite directions on each side of the film. Because the “wires” only have electrode on one side of the film, they do not affect the output of the active PVDF elements. The electrode size of PVDF 1 to PVDF 4 and PVDF 6 is 10 mm × 10 mm. The electrode size of PVDF 5 and PVDF 7 is 20 mm × 10 mm. PVDF 1 to PVDF 4 were designed to investigate the in-plane stress distribution of the cylinder from its center to the edge. PVDF 5 and PVDF 7 were used to check the effect of PVDF size on its output and were located at the symmetrical positions of PVDF 3 and PVDF 1, respectively. PVDF 1 and PVDF 6 are at the same radial distance but at different angles to each other. PVDF 6 was designed to check the effect of sensor directivity compared to PVDF 1. When the PVDF sheet was embedded into the two rubber layers, the mechanical properties of this PVDF layer were uniform so that there were no discontinuities in the stress concentration in horizontal direction. The array was glued using spray contact adhesive. This is because, compared to the individual films in the previous setup, the electrodes on the single sheet have no plastic protection and would be destroyed by the solvents in the Solufix glue. By using spray contact adhesive, this was avoided. During the test, the cylinder was excited by an impact hammer in the normal direction at several different locations on top of the steel block. When the impact force was off center, the axial mode, the shear mode, and the bending mode of the cylinder were all excited (see Figure 7). 

### 3.2. Results of Cylinder 2 and Discussion

**Figure 8:**
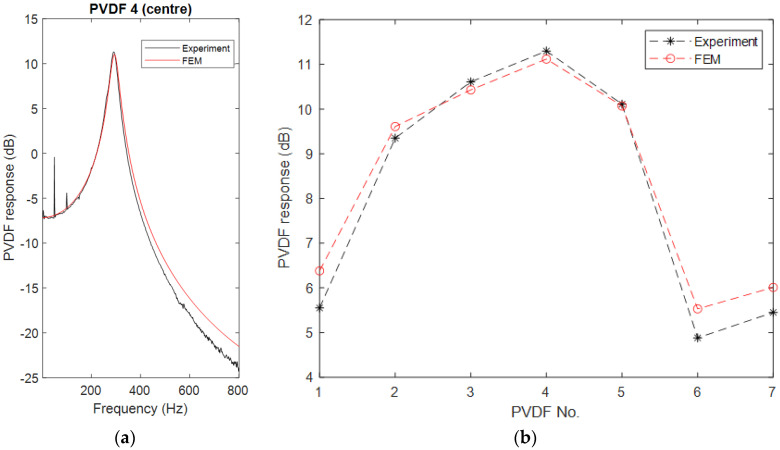
Comparison of PVDF outputs between experiment and FE model: (**a**) frequency response of PVDF 4 and (**b**) the output distribution of the PVDF array at 290 Hz.

In the experiment, a normal impact was first applied at the center of the top steel block. For this case, only the axial mode of the cylinder at 290 Hz was excited. Figure 8 compares the experimental data with the simulated results obtained from the corresponding FE model. Figure 8a shows the frequency response of PVDF 4, while Figure 8b shows plots of the output distribution of the PVDF array at 290 Hz. The maximum difference between the experiment and the FE model is less than 0.8 dB. Although it is larger than the last model of the 5-layer rubber cylinder, the accuracy of the FE model is still satisfactory. The deviation mainly comes from the simplified mechanical properties of PVDF used in the FE model. The mechanical properties of PVDF are also anisotropic [18], so it has different Young’s modulus and shear modulus in its three directions. However, these parameters are not given in the manual, and their measurement has some practical difficulties. Therefore, the PVDF was treated as an isotropic mechanical material in the FE model which leads to some small but inevitable errors. Additionally, it should be noted that this 2-layer rubber cylinder uses a larger PVDF sheet compared with the previous 5-layer rubber cylinder, which may also increase errors in the comparison.

From Figure 8b, several interesting phenomena are observed:

1. In the horizontal direction, the PVDF element closer to the center of the cylinder has a larger output. Both experiment and the FE model indicate that the central element, i.e., PVDF 4, has the largest output and it gradually decreases from PVDF 4 at the center to PVDF 1 close to the edge of the cylinder.
2. The PVDF with the larger electrode areas have smaller outputs compared to their symmetric partners. There are two pairs of PVDF elements whose centers are at symmetrical positions but have different electrode size. One pair is PVDF 1 and PVDF 7, and the other pair is PVDF 3 and PVDF 5. Both the experiment and the FE model demonstrate that the output of the larger elements is slightly less than that of the smaller elements.
3. Although the centers of PVDF 1 and PVDF 6 are at the same radial position and their size is also the same, the output of PVDF 1 is slightly bigger than PVDF 6.

**Figure 9:**
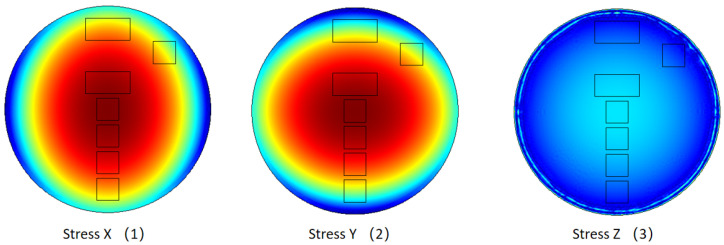
The internal stress field of the PVDF layer in the three directions computed by the FE model.

To understand these phenomena, the internal stress distribution of the PVDF layer is shown in Figure 9. Stress X, Y, and Z correspond to stress applied on the PVDF layer in its x_1_, x_2_, and x_3_ directions, respectively. Firstly, it is clear that the stress distribution is continuous, and no stress concentration appears as was observed when using individual films. Secondly, the in-plane stresses in x_1_ and x_2_ directions are similar but have a 90° phase difference. They are much larger than the vertical stress in the x_3_ direction. No matter in which direction, the stress at the center of the cylinder is larger than that close to the edge. As a result, the PVDF element close to the center rightfully has a larger output. 

**Figure 10:**
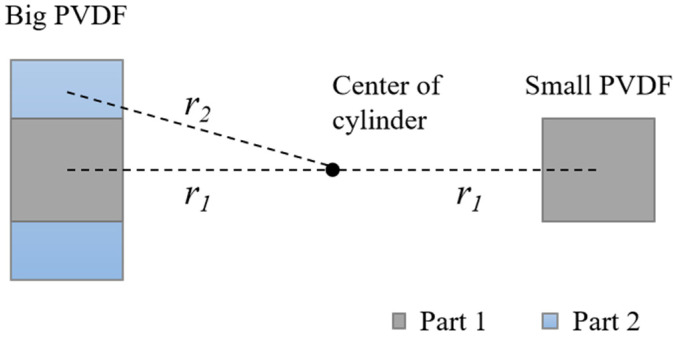
A pair of PVDF elements with different size.

This stress distribution can also explain the second phenomenon. The large PVDF element can be divided into two parts (see Figure 10). Part 1 has the same area to the small PVDF element which also has identical averaged volume stress as the small PVDF. Part 2 has two extra areas at both ends of Part 1. Compared with Part 1, it is farther from the center of the cylinder (r_2_ > r_1_) so that the average volume stress applied on Part 2 is less than that on Part 1. As a result, the overall averaged volume stress of the big PVDF element, which is the mean stress of these two parts, is less than the averaged volume stress of the small PVDF element. For clarity, the averaged volume stresses of PVDF elements computed by the FE model are given in Table 3. As can be seen, for each group, the averaged volume stresses of the big elements are smaller, which results in a smaller output.

The output difference between PVDF 1 and PVDF 6 is mainly due to the directivity of the sensors. The overall in-plane stresses (Stress X + Stress Y) applied on PVDF 1 and PVDF 6 are 6.21 × 10^5^ Pa and 6.29 × 10^5^ Pa, respectively. Although the latter is slightly larger, the output of PVDF 6 is smaller. From Table 3, it is clear that the tangential stress ($\bar{\tau_{11}}$ for PVDF 1) is larger than the radial stress ($\bar{\tau_{22}}$ for PVDF 1). The PVDF used in this paper is anisotropic. Its sensitivity in x_1_ direction is 5 times greater than that in x_2_ direction (see Table 1). For PVDF 1, its x_1_ direction is along the tangential direction which maximizes its output. However, the x_1_ direction of PVDF 6 has a 45° angle to the tangential direction which reduces its output. 

**Figure 11:**
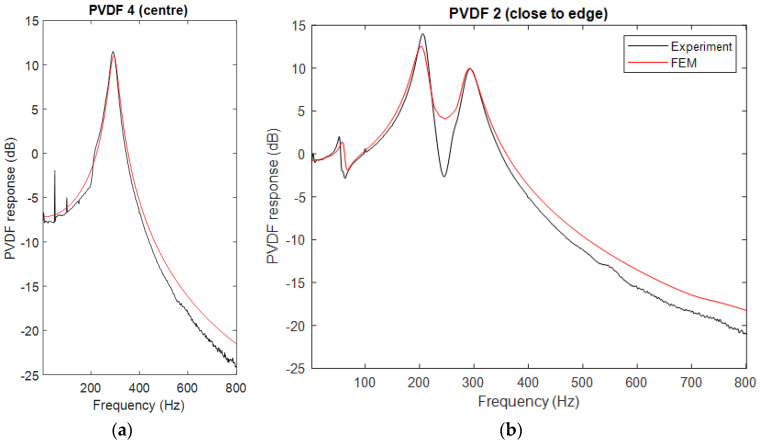
Frequency response of (**a**) PVDF 4 and (**b**) PVDF 2 when the impact is 20 mm away from the center.

When the excitation position is moved off-center, all three modes of the cylinder were excited. Figure 11 displays the frequency responses of PVDF elements when the impact was 20 mm away from the center along the array direction. The measured natural frequencies of the shear, bending, and axial modes are at 60 Hz, 209 Hz, and 290 Hz, respectively. The FE model shows a good agreement with the experimental data. Both results indicate that although all three modes of the cylinder are excited, the central PVDF element, PVDF 4, can only sense the axial mode. This is because, for the shear and bending modes, PVDF 4 is located at the central point of these antisymmetrical modes. Therefore, the stress applied to one half of PVDF 4 is positive (compression) and on the other half is negative (expansion). They, therefore, cancel each other so that the averaged stress due to these modes is zero. For the rest of the PVDF elements, the three modes can be measured. The element farther from the center of the cylinder is more sensitive to the shear and bending modes.

## 4. Conclusions

This paper reports a series of the experimental and theoretical works on embedded PVDF. Two rubber cylinders with PVDF arrays were tested experimentally, and their corresponding FE models were established. Through this investigation, the following key points were found:

- The output of embedded PVDF is significantly affected by the elastic material around it. Even when the excitation is only in the normal direction, the Poisson effect of the elastic material can generate large in-plane stress which dominates the output of embedded PVDF.
- The output of embedded PVDF is determined by the averaged volume stress applied on it. The size of embedded PVDF does not directly link to its sensitivity. Increasing the size may even lead to an opposite result. Therefore, it is particularly important to carefully choose the size of embedded PVDF for various applications. In fact, if the stress distribution of the structure can be determined, a higher sensitivity can be obtained by placing a small-sized PVDF at the antinode of the stress field.
- To improve the sensitivity of embedded PVDF, its directivity should be considered. For anisotropic PVDF, the sensitivity in the x_1_ direction can be tens of times that in the x_2_ direction. Therefore, in practical applications, it should be ensured that the x_1_ direction of PVDF is aligned with the direction of high stress.
- When the embedded PVDF is located at the center point of an asymmetrical mode of the elastic material, the positive and negative stress applied on it will cancel with each other so that the sensitivity to this mode significantly decreases. This location should be avoided if this mode is important in the measurement. On the other hand, this phenomenon can also be exploited to avoid interference from an unwanted mode.

In conclusion, this investigation provided some useful information for further understanding the working mechanisms of embedded PVDF in elastic materials, which may throw some light on the design of new PVDF sensors.

## Figures and Tables

**Table 1:** Parameters used in FE model.

**Rubber**	Density (kg/m^3^)	Young’s Modulus (Pa)	Loss Factor	Poisson’s Ratio
	1050	2.3 × 10^6^	0.15	0.495
**PVDF**	Density (kg/m^3^)	Young’s modulus (Pa)	Loss factor	Poisson’s ratio
	1780	4.6 × 10^9^	0	0.35
	g_31_ (Vm/N)	g_32_ (Vm/N)	g_33_ (Vm/N)	
	0.21	0.042	−0.30	

**Table 2:** Averaged volume averaged stresses applied on PVDF elements at 87 Hz.

	$\bar{\tau_{11}}$	$\bar{\tau_{22}}$	$\bar{\tau_{33}}$
**PVDF 1**	4.36 × 10^5^ Pa	2.57 × 10^5^ Pa	−4.30 × 10^3^ Pa
**PVDF 2**	6.60 × 10^5^ Pa	3.86 × 10^5^ Pa	−5.24 × 10^3^ Pa
**PVDF 3**	6.64 × 10^5^ Pa	3.92 × 10^5^ Pa	−5.33 × 10^3^ Pa
**PVDF 4**	4.47 × 10^5^ Pa	2.62 × 10^5^ Pa	−4.54 × 10^3^ Pa

**Table 3:** Averaged volume averaged stresses applied on PVDF elements at 290 Hz.

	Group 1		Group 2	
	PVDF 1 (Small)	PVDF 7 (Big)	PVDF 3 (Small)	PVDF 5 (Big)
$\bar{\tau_{11}}$	3.56 × 10^5^ Pa	3.30 × 10^5^ Pa	4.98 × 10^5^ Pa	4.63 × 10^5^ Pa
$\bar{\tau_{22}}$	2.65 × 10^5^ Pa	2.49 × 10^5^ Pa	4.90 × 10^5^ Pa	4.56 × 10^5^ Pa
$\bar{\tau_{33}}$	−9.60 × 10^3^ Pa	−9.23 × 10^5^ Pa	−1.72 × 10^4^ Pa	−1.60 × 10^4^ Pa

## Data Availability

The data presented in this study are available on request from the corresponding author.
